# Fabrication of k-Carrageenan/Alginate/Carboxymethyl Cellulose basedScaffolds via 3D Printing for Potential Biomedical Applications

**DOI:** 10.3390/polym16111592

**Published:** 2024-06-04

**Authors:** Cristina Stavarache, Adi Ghebaur, Andrada Serafim, George Mihail Vlăsceanu, Eugeniu Vasile, Sorina Alexandra Gârea, Horia Iovu

**Affiliations:** 1 Advanced Polymer Materials Group, National Polytechnic University of Science and Technology Bucharest, 1-7 Gh. Polizu Street, 011061 Bucharest, Romania; cristina.stavarache@upb.ro (C.S.); adi.ghebaur@upb.ro (A.G.); andrada.serafim0810@upb.ro (A.S.); george.vlasceanu@upb.ro (G.M.V.); sorina.garea@upb.ro (S.A.G.); 2 C.D. Nenițescu” Institute of Organic and Supramolecular Chemistry, 202-B Spl. Independentei, 060023 Bucharest, Romania; 3 Faculty of Medical Engineering, National University for Science and Technology Politehnica Bucuresti, 1-7 Gh. Polizu Street, 011061 Bucharest, Romania; 4 Department of Science and Engineering of Oxide Materials and Nanomaterials, Faculty of Applied Chemistry and Material Science, National Polytechnic University of Science and Technology Bucharest, 1-7 Gh. Polizu Street, 011061 Bucharest, Romania; eugeniuvasile@yahoo.com; 5 Academy of Romanian Scientists, 54 Splaiul Independentei, 050094 Bucharest, Romania

**Keywords:** vitamin B1, polysaccharide scaffolds, drug delivery systems

## Abstract

Three-dimensional (3D) printing technology was able to generate great attention because of its unique methodology and for its major potential to manufacture detailed and customizable scaffolds in terms of size, shape and pore structure in fields like medicine, pharmaceutics and food. This study aims to fabricate an ink entirely composed of natural polymers, alginate, k-carrageenan and carboxymethyl cellulose (AkCMC). Extrusion-based 3D printing was used to obtain scaffolds based on a crosslinked interpenetrating polymer network from the alginate, k-carrageenan, carboxymethyl cellulose and glutaraldehide formulation using CaCl_2_, KCl and glutaraldehyde in various concentrations of acetic acid. The stabile bonding of the crosslinked scaffolds was assessed using infrared spectroscopy (FT-IR) as well as swelling, degradation and mechanical investigations. Moreover, morphology analysis (µCT and SEM) confirmed the 3D printed samples’ porous structure. In the AkCMC-GA objects crosslinked with the biggest acetic acid concentration, the values of pores and walls are the highest, at 3.9 × 10^−2^ µm^−1^. Additionally, this research proves the encapsulation of vitamin B1 via FT-IR and UV-Vis spectroscopy. The highest encapsulation efficiency of vitamin B1 was registered for the AkCMC-GA samples crosslinked with the maximum acetic acid concentration. The kinetic release of the vitamin was evaluated by UV-Vis spectroscopy. Based on the results of these experiments, 3D printed constructs using AkCMC-GA ink could be used for soft tissue engineering applications and also for vitamin B1 encapsulation.

## 1. Introduction

Over the years, an increased interest in using natural polymers as inks to assemble complex biomedicals objects and implants [1] has been observed. Layer-by-layer deposition through extrusion-based 3D printing of the biomaterial as well as accurate cell placement [2,3] represent one of the preferred routes to obtain functional scaffolds for various applications. Moreover, customized biomedical scaffolds may be obtained to perfectly match the damaged region in the patient’s body, having been manufactured through a combination of different techniques, i.e., using the images from magnetic resonance imaging (MRI) or computed tomography (CT) and digitally 3D printing designs [4]. Additionally, due to the manufacturer’s ability to modify oral dose forms, 3D printing is becoming a more commonly employed technique in the pharmaceutical sector. As a result, it facilitated the development of targeted drug delivery systems with specific features such as permeability, pore size, hydrophobicity, hydrophilicity or functionality. By combining these properties, the possibility of fabricating a scaffold that would synergistically carry a medicine to a specific site of the body while helping in tissue regeneration becomes more attainable [5,6,7]. For of the ink to be used, several characteristics must be tailored to obtain a highly functional scaffold that would mimic the native tissue, ranging from the cell compatibility perspective to polymer type and crosslinking the process with the physiological and mechanical attributes [8,9,10,11,12]. Furthermore, the biomaterial from which the suitable ink is made should be biocompatible, non-toxic, biodegradable and printable [10,12].

Printability refers to a material’s ability to be dispensed in self-standing filaments with the required dimensions for a given purpose [8,9]. Therefore, the printable ink should have gel-like features, high thixotropicy [12] leading to a stabile structure that will resist mechanical forces and would not shrink considerably during drying [13] and should maintain cell viability after printing [9]. Moreover, the interfacial strength between material layers should be robust enough, thus preventing the delamination both during and after printing [14].

Polysaccharide-based hydrogels are attractive to work with in this particular field due to their quality of being biodegradable and being found in abundance in nature, and therefore, these two simple factors bring the cost down; additionally, they are biocompatible and have suitable pharmacological properties [15,16,17]. Polysaccharides which are used for the encapsulation of drugs can establish a protective layer between the active ingredient formulation and its environment which improves the medication’s stability, prolongs its pharmaceutical activity [16] and reduces its side effects [18]. However, their resistance to mechanical stress is limited and their specialized chemical and biological characteristics led to the development of novel multi-component inks such as interpenetrated networks (IPNs) [11,17,19,20,21].

Alginate [22], chitosan [23], pectin [24], carboxymethyl cellulose [2], agarose [25] and k-carrageenan [26] are often used for their biological and physical properties that are vital for obtaining suitable scaffolds [16,27]. 3D printed hydrogels made of chitosan and pectin were assessed for lidocaine delivery in wound treatment; the results indicated that these materials represent proper candidates for wound dressings [24].

Alginates (Alg), k-carrageen (kCG) and carboxymethyl cellulose (CMC) are becoming increasingly used in regenerative medicine and drug delivery systems developed to formulate inks that completely form an interpenetrating polymer network (IPN) through physically and chemically crosslinking which gives them thermal stability, better mechanical properties, chemical resistance and swelling degree improvement [16,17,19]. The obtained biomaterials, even if they are semi–IPNs or subsequent–IPNs or subsequent semi–IPNs, possess all of the characteristics of such materials as well as non-immunogenicity, high stability and biological compatibility together with non-cytotoxicity and being made of low-price materials [10,17]. Alginate is an anionic natural polymer extracted from marine brown algae, composed of two structural units of β-D-mannuronic acid named M block and α-L-guluronic acid named G block [11,16,20]. However, alginate solutions fail to create stable 3D printing structures because of their rheological behavior [8,20]. Alginate has interesting properties that solve this limitation. It forms gels under light conditions at room temperature with divalent cations, mostly with Ca^2+^, and also by interacting with cells or creating polyelectrolyte complexes (PE) [28] to strengthen stability of the alginate hydrogel, to enhance cell adhesion and the rheological and mechanical properties of the 3D printed structure [20,25,29,30,31]. To this end, Alg was mixed with gelatin [20,32], collagen [3], carboxymethyl chitosan [33], k-carrageenan [34], cell adhesion ligands [31], methyl cellulose [14], nanoparticle-based systems and some additives like hydroxyapatite, silica and gel- and film-based materials [31,35]. Also, a ternary mixture composed of alginate/chitosan and nano hydroxyapatite was formulated by Ali Sadeghianmaryan [36] to reinforce the hydrogel scaffolds for applications in cartridge regeneration due to its increased mechanical and biological features. Another three-component bio-ink made from alginate, gelatin and cellulose provides a suitable environment for cell development and proliferation [4].

Cellulose-based formulations show significant potential in 3D bioprinting. CMC, another derivate of cellulose, the most abundant biopolymer in nature, can also be employed as a component in bioink for many biomedical applications as a consequence of its properties; it is highly hydrophilic and an excellent viscosity changer, possesses good gelation conditions as well as biocompatibility and matrix-formation ability, and it is biodegradable [37,38,39,40]. Interestingly, CMC has been demonstrated to stimulate the chemotactic migration of cells on its own and to promote cell migration in response to soluble stimuli [39]. Additionally, CMC is used in numerous industries like pharmaceutics, food and waste management, cosmetics and paper, since it easily crosslinks and due to its thickening properties and better swelling behavior [41]. Also, cellulose and its derivatives were utilized as an organic template to create several unique and adaptable electromagnetic (EMI) shielding materials [42]. A composition of these two natural polymers, Alg and CMC, was developed by Habib [2] to obtain an improved printing ink which has better rheological characteristics. Both have similar rapid crosslinking conditions, and they form hydrogels in the presence of Ca^2+^. Moreover, the Alg/CMC formulation was used in wound healing because it promotes cellular attachment and migration, is non-toxic, and has proper viscosity and elasticity qualities and excellent gelation conditions that can be easy to manage and that can produce superior ink for printing [38,43]. The analgesic drugs diclofenac and lidocaine were incorporated in this hydrogel, and this did not significantly influence the printability of the ink [37]. Mixing Alg and CMC with synthetic nanosilicate clay delivered a good fidelity and better extrusion, and by incorporating immortalized hMSCs, the 3D printed scaffolds showed a 70% cell viability for over 21 days in biological studies [44].

k-Carrageenan (kCG) is a linear sulfated natural polymer with excellent characteristics that makes it an excellent option for tissue engineering applications and in the pharmaceutical field [45,46]. It is a hydrophilic biopolymer with one ester sulfate group in the backbone’s structure which consists of repetitive 3-linked-α-D-galactopyranose and 4-linked-β-D-galactopyranose units, and the sulfate groups are what provides cell adhesion and proliferation [34,45]. kCG has also been shown to hinder inflammatory reactions due to its negatively charged carboxyl and sulfate groups. Furthermore, kCG’s structure resembles mammalian glycosaminoglycans that can be found in an extracellular matrix as a major component, and has powerful attachment with proteins and enzymes [34,45,47]. Besides these advantages, kCG is used for gelling and thickening and for its mechanical properties in food and pharmaceutical applications. The gelling process occurs through two mechanisms: (1) at low temperature, even at room temperature, because of the transition from random coil to a double helix of polymeric chains of kCG, and (2) ionically, by interacting with mono- and divalent cations (K^+^, Na^+^, Ca^2+^) [34,48,49]. Due to its biocompatibility and its important antioxidant, anticoagulant, anti-inflammatory and anti-allergic properties and its availability in nature, kCG is used as a carrier for the controlled release of drug delivery carriers and as a component in ink formulations for 3D printing. Its viscosity, which can be customized by variating concentration, temperature and ion concentration, makes kCG an appropriate candidate for bioink compositions [34,46,50]. kCG was combined with alginate [34], methylcellulose and poly (3,4 ethylenedioxythiophene):poly(styrenesulfonate) [51], gelatin [52], methylcellulose/cellulose nanocrystal [53] and with nanosilicates [25] to enhance the thixotropic behavior of the ink. Moreover, methacrylate-modified kCG and methacrylated gelatin were used by L. Tytgat [54] as a biomaterial ink suitable for applications in adipose tissue engineering.

The present work proposes a ternary polysaccharide-based composition that provides an improved printable hydrogel ink to obtain an advanced and unique biomaterial in 3D printing suitable for the manufacturing of the alginate-k-carrageenan-carboxymethyl cellulose-glutaraldehyde scaffolds (AkCMCG) for biomedical applications. To the best of our knowledge, there is no reported research on the subject of preparing this type of three-component ink for 3D printing technology.

The preparation of the polysaccharide mixture and the optimal concentration of these three components were investigated and rely on the knowledge from the previous work of our group [55] in order to obtain a suitable ink for 3D printing. kCG/Alg IPN gel beads were obtained successfully for the encapsulation of ketoprofen [18] by our group, and taking into consideration the results from testing the kCG/Alg ink in 3D printing, CMC was chosen to be added to the mixture due to its properties that were mentioned before. All three components have similar crosslinking conditions and can have a synergetic effect. Also, the influence of the acetic acid concentration on the structural stability of the printed samples was assessed.

Additionally, this study shows the incorporation of vitamin B1 and its release into and from the new AkCMCG scaffolds for the first time in this kind of carrier. Thiamine, also known as vitamin B1, is one of the most essential micronutrients for human health; it is a coenzyme precursor used in the metabolism of carbohydrates and a deficiency in vitamin B1 may result in beriberi illness, which occurs because of the damaging of the glucose oxidation process and the inadequacy of pyruvate oxidation [56,57]. This vitamin helps combat stress, improve immunity, prevents neurological problems and enhances memory and mood [57]. These three natural carbohydrates were individually used by Marta Tsirigotis-Maniecka [16] as protective materials for the encapsulation of cranberry fruit extract to preserve its antioxidant activity, proving that sodium alginate and carboxymethyl cellulose were suitable for the delivery of cranberry extract while enhancing long-term storage stability.

## 2. Materials and Methods

### 2.1. Materials

Medium-molecular-weight sodium alginate (Alg) (Alginic acid sodium salt from brown algae), k-Carrageenan (kCG) (predominantly k and lesser amounts of λ carrageenan), potassium chloride (KCl), potassium phosphate monobasic (KH_2_PO_4_), sodium hydroxide pellets (NaOH), acetic acid glacial (AA) and thiamine hydrochloride (VitB1) were purchased from Sigma-Aldrich, St. Louis, MO, USA; calcium chloride (CaCl_2_) was purchased from Carl Roth, Karlsruhe, Germany; glutaraldehyde (GA) 25 wt% solutions in water from Alfa-Aesar, Kandel, Germany; and carboxymethyl cellulose sodium salt (CMC), M.W. 90,000 from Acros Organics, Fair Lawn, NJ, USA.

### 2.2. Preparation of Polysaccharide Ink

To prepare the formulations for printing, different amounts of kCG and Alg were put together in a 0.5% glutaraldehyde (GA) solution, a widely used crosslinker, to obtain a biopolymer mixture of 3 wt% with weight ratio of Alg/kCG = 1:1, under magnetic stirring at 85 °C. When the Alg/kCG blend was completely homogenized, CMC powder (1 wt%) was gradually added into the heated composition. Finally, after the complete dispersion of CMC, the polysaccharide combination was stored at 4 °C overnight.

### 2.3. Rheological Characterization of Precursors

The viscoelastic behavior of the three-component formulations with and without GA was assessed using a Kinexus Pro rheometer (Malvern, Worcestershire, UK) equipped with a Peltier element for precise temperature control. To evaluate the rheological properties of the biopolymer mixture, a parallel plate geometry was used (upper plate diameter of 20 mm). The viscosity (η) of the precursor solutions was measured in the shear rate interval 0.01–10 s^−1^ at 25 °C. To protect the samples from drying, a water lock was used during tests. The recovery properties of the polysaccharide solutions were measured in order to examine how the inks’ structure responds during the 3D printing process. The test involves three phases: in the first step, a very low shear rate of 0.1 s^−1^ was applied for 60 s, then, in the second step, a shear rate of 100 s^−1^ was tested for 10 s. In the final phase, the materials were examined again at a very low shear rate of 0.1 s^−1^ for 300 s.

### 2.4. 3D Printing of the Scaffolds

The polysaccharide-based scaffolds were fabricated using the Direct Dispenser DD135N print-head of the four-head 3D Discovery Bioprinter from RegenHU, Ltd, Villaz-St-Pierre, Switzerland, and the layer-by-layer deposition technique was carried out to obtain the 3D structures. Prior to loading the cartridge, formulation was heated and then slowly poured into the 3-mL syringe with a disposed cylindrical nozzle and a 25 gauge (ø 0.25 mm, needle length 6.35 mm) needle and was left to cool down for the thermal gelation of the composition to take place at ambient temperature. The printability trails started by printing a continuous, single-layer strand structure of the material, and then a series of tests were conducted to optimize printing parameters by varying different pressures and printing speeds. All trials were carried out at room temperature. The obtained 3D printed depositions of Alg-kCG-CMC-GA formulation (termed AkCMCG), designed as a cylinder with grid structure, were crosslinked by immersing it in a mixture of CaCl_2_:KCl = 1:1 (volumetric ratio) of 0.5M with 10% GA for two hours. The 10% GA solutions were prepared in different acetic acid (AA) concentrations: 1%, 2% and 3%. The crosslinking baths were composed by mixing equal volumes of GA with chloride solutions. To simplify, the 3D printed scaffolds are termed AkCMCG-1A, AkCMCG-2A and AkCMCG-3A for the samples that were crosslinked with 10% GA prepared in 1% AA, 2% AA and 3% AA together with the chloride mixture. At the end of the crosslinking period, the scaffolds were thoroughly rinsed with deionized water and freeze-dried at 0.03 mbar with temperature of [−35 °C:+35 °C] (Alpha 2–4 LSC Basic, Martin Christ, Osterode am Harz, Germany). The same protocol was used for obtaining scaffolds used for the assessment of the drug entrapment and release profile.

**Figure 1:**
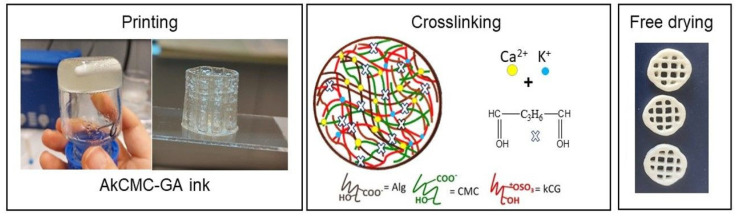
Schematic workflow representation for manufacturing the 3D printed scaffold (sample diameter, 14 mm and line space 3.5 mm) which includes printing, crosslinking and freeze−drying.

Figure 1 illustrates the obtained ink, the printing process and the obtained scaffold before and after crosslinking.

### 2.5. Morphological Analysis

Morpho-structural characterization of 3D printed scaffolds was carried out through micro-CT (µCT) and scanning electron microscopy (SEM).

Freeze-dried 3D printed objects AKCMCG-1A, AKCMCG-2A and AKCMCG-3A were scanned with Bruker µCT 1272 high-resolution equipment with the following parameters pre-set: no filter, 50 kV source voltage, 150 µA current intensity, 450 ms exposure per frame. The acquisition process was carried out during a 180° rotation of the sample, with a rotation step of 0.2°, with an average of 4 acquisitions for each position. The scanning resolution (image pixel size) was set at 10 µm. 3D reconstruction was performed in Bruker NRecon 1.7.1.6 software (Kontich, Belgium), while quantitative analysis and pore reconstruction as solid objects were performed in Bruker CTAn 1.17.7.2 software (Kontich, Belgium). Solid sample and pore datasets that resulted were rendered (both separately and simultaneously) in Bruker CTVox 3.3.0.0 (Kontich, Belgium).

The surface morphology of the 3D printed constructs was assessed through Quanta Inspect F50 Scanning Electron Microscopy (SEM) (FEI, Hillsboro, OR, USA). The freeze-dried samples were coated under vacuum with a thin gold film prior to testing.

### 2.6. Swelling Behavior of the 3D Printed Hydrogel Structures

The swelling measurements of the dried 3D printed constructs were determined at 37 °C by independently incubating three scaffolds of each composition in plastic tubes containing PBS pH = 7.4 using a thermostat water bath from GFL 1083, Kischer Biotech, Steinfurt, Germany. The PBS solution was prepared in our laboratory using NaOH and KH_2_PO_4_ with pH of 7.4 at 37 °C. The swelled scaffolds were withdrawn from the testing media at predetermined time intervals and weighed after blotting residual liquid on the surfaces with filter paper.

The swelling ratios (SR, %) of the 3D printed samples at any time were calculated using Equation (1) [58]:

Swelling rate (SR, %) = (Wt − W0)/W0 × 100(1)

where W0 is the weight of the dried specimen (before being immersed in PBS) and Wt is the wet weight of specimen at time t.

The maximum swelling degree (MSD) was calculated using Equation (2) [24]:

MSD, % = (We − W0)/W0 × 100(2)

where We is the weight of the specimen at hydration equilibrium.

Three samples of each batch were used for these tests.

### 2.7. Structural Stability

To figure out the structural stability of the 3D printed scaffolds, the samples were placed by immersing the dried constructs individually in PBS, pH = 7.4 at 37 °C in a thermostat water bath from GFL 1083, Kischer Biotech, Steinfurt, Germany. The PBS solution was changed throughout the experiment. The construct degradation degree was assessed gravimetrically using Equation (3). The maximum weight of the swollen sample was used as the initial weight.

Degradation degree (DD, %) = (Wmax − Wt)/Wmax × 100(3)

where Wmax represents maximum weight of the swollen sample and Wt is the recorded value of the scaffold after immersion at different periods of time. The specimens were dabbed and weighted. The experiment was performed in triplicate.

### 2.8. Mechanical Properties of the 3D Construct

The mechanical characteristics of 3D printed AkCMCG-1A, AkCMCG-2A and AkCMCG-3A samples were determined using a Brookfield CT3 texture analyzer equipped with a TA4/1000 compression accessory. Cylindrical 3D printed constructs ~15 mm in diameter and ~4 mm in height were hydrated to equilibrium and placed on the bottom plate of the equipment. Uniaxial compression was applied up to a deformation of 90% using a compression speed of 0.1 mm/s. A stress deformation curve was plotted using the dedicated software (TexturePro CT V1.8 Build 31). The compression modulus (E’) was read on the curve at a strain of 2%, in the linear region. The tests were performed at room temperature, in triplicate. The results are presented as means of the registered values.

### 2.9. Drug Entrapment

The encapsulation efficiency (EE %) of VitB1 was determined by immersing freeze-drying scaffolds into an aqueous solution of 1mg/mL until the hydration equilibrium was reached, and then the residual solvent was analyzed through a UV-Vis spectrophotometer at λ = 242 nm, UV 3600 Shimadzu, Kyoto, Japan. The VitB1 assay was assessed using a calibration curve prepared with concentrations of vitamin between 0.00004 and 0.0002 mg/mL. The measurements were made in triplicate.

EE (%) = (Loaded amount of VitB1)/(Total VitB1) × 100(4)

### 2.10. Fourier Transform Infrared (FT-IR) Spectroscopic Studies

The structural study of each composition of AkCMCG 3D printed specimens with and without vitamin B1 (VitB1) was carried out thought a Bruker Vertex 70 FTIR spectrometer (Bruker, Billerica, MA, USA) equipped with an attenuated total reflectance (ATR) accessory. The spectra were acquired in the range of 600–4000 cm^−1^ and with 32 scans at room temperature.

### 2.11. Dissolution Testing

For the drug release studies of the VitB1, a fully automated dissolution bath USP Apparatus 1 (708-DS Agilent Agilent Technologies, Inc, Santa Clara, CA, USA) connected with an auto controlled multi-channel peristaltic pump (810 Agilent) to a UV-Vis spectrophotometer (Cary 60) with 1 mm flow cell was employed. The VitB1–loaded 3D printed scaffolds were placed in a dialysis membrane bag filled with 5 mL of PBS, pH = 7.4 solution and then the dialysis membrane bags were put in 200 mL buffer solution at 37 °C, and the spindles’ rotation speed was set at 75 rpm. At predetermined time intervals, volumes of test media were automatically extracted, and the amount of drug released was determined using UV-Vis spectrophotometer at λ = 242 nm.

## 3. Results

### 3.1. Rheological Characterization of the Precursors

To enhance the printing performance, the precursor requires excellent thixotropic behavior, and after printing the material must have an acceptable recovery period. It is important for printable ink to have an optimized viscosity during printing. At the same time, the material subjected to shearing must also exhibit dimensional stability after the shear force is removed and acceptable mechanical properties after printing [59].

To investigate the rheological properties of the material subjected to printing, shear viscosity studies were performed on the Alg-kCG-CMC solution and on Alg-kCG-CMC with GA at 25 °C. The 3% Alg and kCG solutions and 1% CMC solution were used as a control.

**Figure 2:**
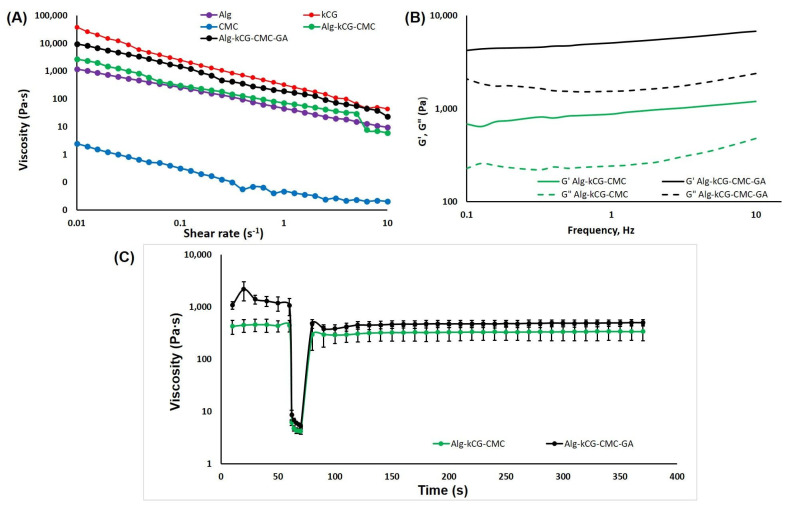
(**A**) The dependence of viscosity on the shear rate at 25 °C; (**B**) The dependence of storage modulus G′ and loss modulus G″ on the frequency of the studied solutions; (**C**) The creep and recovery behavior of the Alg-kCG-CMC and Alg-kCG-CMC-GA mixtures at different shear rates (0.1 s^−1^ and 100 s^−1^) at 25 °C.

As observed in Figure 2A, all of the tested solutions display a shear-thinning conduct; their viscosity dropped as the shear rate increased during the tested shear rate domain. This characteristic of the formulations demonstrated the feasibility of injecting the ink and indicated a non-Newtonian behavior for the well-blended inks, which is necessary to ensure consistent flow through a convergent nozzle when subjected to external pressure [42]. The CMC solution indicates a considerably lower viscosity than the other tested solutions which are more concentrated. In contrast, the kCG solution has the highest viscosity due to the biopolymer’s thermo-reversible properties, and even at room temperature, it is able to form a gel-like composition [48]. We found that the curves registered for the mixtures of the polysaccharides with and without added GA are placed between the Alg and kCG solutions. The presence of GA in the three-component formulation was found to increase viscosity, the curve being very near to that of the kCG solution. We attributed this behavior to the formation of hydrogen bonds between the –OH groups of the natural polymers [60] and possibly to the physical bonds established between the GA and the natural macromolecules.

The frequency sweeps performed in the range of 0.1–10 Hz offer information on the inks’ stability. To evaluate the effect of CaSO_4_ on the mechanical strength of hydrogels, Kim et al. conducted a frequency sweep test on a 2% alginate solution with various CaSO_4_ concentrations [34]. The results are presented as logarithmic graphs in Figure 2B.

As depicted in Figure 2B, these two formulations, AkCMC and AkCMC-GA, are influenced by the frequency and exhibit a rise in G′ and G″ as the frequency increases. Moreover, both samples display a gel-like behavior, with the loss modulus, G′, predominant over the storage modulus, G″, and improved stability in the tested frequency domain. Additionally, adding GA to the AkCMC composition increased the G′ and G″ values compared with the AkCMC mixture.

To monitor the viscosity change of the prepared inks based on cellulose nanofibers and photo-crosslinkable biopolymers after extrusion, a viscosity recovery test was conducted by Wang et al. Also, to study the intermolecular interactions between the cellulose nanofibers with gelatin methacryloyl or methacrylated galactoglucomannan, quantitative rheological measurements were carried out [61].

Figure 2C shows the creep and recovery behavior of the Alg-kCG-CMC and Alg-kCG-CMC-GA formulations. The thixotropic behavior test begins at a low shear rate of 0.1 s^−1^ for 60 s to mimic the stationary condition of the ink before extrusion; following that, a second step at a shear rate of 100 s^−1^ was applied for 10 s to simulate the state of the material during extrusion through a needle. Finally, in the third step, the same parameters were used (shear rate of 0.1 s^−1^) as in the first step but for a longer time, 300 s, in order to observe the recovery of the ink structure after the shear rate is removed at the end of the printing process [34,62].

As illustrated in Figure 2C, the viscosity of the two precursors reduced dramatically at high shear rates and quickly recovered at low shear rates. For the Alg-kCG-CMC sample, the results indicated a recovery time of 20 s with a 69% recovery of the initial viscosity and a 79% recovery at the end of the trial. For the Alg-kCG-CMC-GA formulation, the results exhibited a recovery time of 10 s with a 44% recovery of the initial viscosity and a 46% recovery at the end of the test. Its viscosity decreased from 1083.33 Pa∙s at 0.1 s^−1^ (initial value) to 5.30 Pa∙s at 100 s^−1^ (the last value from the second step), and then, in 10 s, rapidly increased to 480.87 Pa∙s. These rheological results indicated that the formulations can be used for 3D printing due to the inks’ rapid and reversible viscosity response; they can be easily extruded and then rapidly regain their mechanical strength to maintain the next layer [59].

### 3.2. Scaffolds Fabrication

Three plant-derived carbohydrates were chosen because their structures have various functional groups (–COOH, –OH, –OSO_3_H) that can engage in ionic crosslinking and generate physically stable gels. Also, to optimize the printability of gellan gum, biopolymers such as methyl cellulose and sodium alginate were incorporated in the formulation by Qin Chen et al. in their research [63]. To fabricate the printed scaffolds, the direct dispenser print-head of the 3D DiscoveryTM bio-printer was used, and a layer-by-layer fashion of filaments was engaged. The printability of the proposed formulation was tested in order to dispense a continuous filament, and also the printing parameters were optimized through multiple trials. The composition was poured into a 3 mL cartridge and dispensed pneumatically using metallic needles with variable inner diameters (0.20 mm and 0.25 mm). At first, the printing speed (4, 6 and 7 mm/s), and the extrusion pressure (160–200 kPa) were adjusted on a 2D design generated by a G-code from the AutoCAD software (BioCAD 1.1). A 3D structure with multiple layers was manufactured so that the structural and mechanical strength of the printed construct could be evaluated.

**Figure 3:**
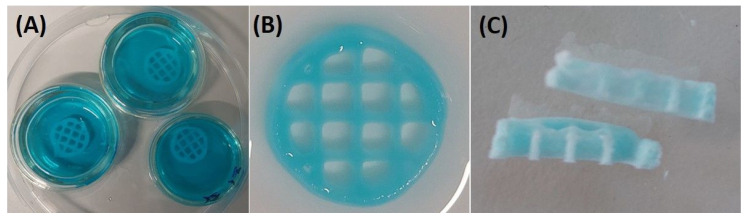
The 3D printed constructs’ (sample diameter 14 mm and line space 3.5 mm) crosslinking procedure. (**A**) The incubation of 3D printed layer-by-layer depositions in the blue food-dyed crosslinking solution; (**B**) the scaffold after proper washing; (**C**) the freeze-dried sample.

In this research, the AkCMCG ink filaments were continuous and homogeneous, as shown in (Figure 3A) and the 3D layer-by-layer deposition was able to hold up to 60 layers without collapsing. The printing parameter optimization is presented in Table 1 and the manufactured layer-by-layer objects are displayed in Figure S1 from the Supplementary Material.

To assess the shape fidelity, stability and repeatability of the 3D printed object, nine scaffolds (16 layers, sample diameter: 14 mm and line space 3.5 mm) were obtained using the same optimized parameters of 4 mm/s for printing rate and 165 kPa for extrusion pressure. Also, the real scaffolds’ height was compared with the theoretical (ht = 3.5 mm for 14 layers and ht = 4 mm for 16 layers) from the AutoCAD software as shown in Figure S2 from the Supplementary Material.

After the 3D scaffolds were obtained, the 3D constructs were crosslinked for two hours through incubation in a mixture of equal volumes of CaCl_2_ 0.5 M and KCl 0.5 M aqueous solutions and 10% GA prepared in various AA concentrations. The volumetric ratio between the ionic solution (CaCl_2_ + KCl) and the 10% GA solution was 1:1. After the crosslinking process was completed, the samples were thoroughly washed and then freeze-dried. The 3D printed depositions had proper stability structures because of the thermo-reversible property of kCG, a unique feature that makes the structures support themselves [25,64,65], and also due to the thickening characteristic of CMC [2]. Additionally, the constructs obtained using the extrusion-based technique requires a fast gelation. On one hand, the reinforcement of the scaffolds is achieved by crosslinking the ink components through the obtaining of the interpenetrating ionic network (IPN) between kCG, CMC and Alg in the presence of the Ca^2+^ and K^+^. The rapid crosslinking of Alg is achieved via the ionic interaction of the –COO^−^ group from the G and M blocks of two side-by-side polymer chains with Ca^2+^, which creates ionic inter-chain bridges [2,66]. Ca^2+^ acts as chelating center that contributes to the stabilization of these interactions [4]. Similarly, the cellulosic component forms, in the presence of Ca^2+^ ions, a calcium complex due to the existence of carboxyl groups in its structure [2]. kCG engages in the crosslinking process via the ionic interaction between the K^+^ ions with sulfate groups from the polysaccharide’s structure, generating the ordered double-helix structure of the chains via coil to helix transition with K^+^ and Ca^2+^, but the interaction also changes kCG to a rigid hydrogel [46,64,67].

The physical crosslinking was achieved through the diffusion method [68] (Figure 3). Adding CMC to the Alg/kCG blend could improve the mechanical strength of the printed scaffold [69]. Furthermore, it is simple to add growth factors to cellulose materials, which will promote maximal vascularization, reduce the early immunological response and allow specific cell types to penetrate [70].

On the other hand, the augmentation of the hydrogels’ stability was finalized by additionally crosslinking with GA, which reacts with the hydroxyl groups of the polysaccharides, but only in acidic conditions [4,71]. In this research, the effect of the different concentrations of the acidic agent on the hydrogel strengthening was studied in this manner for the first time, to our knowledge. As a consequence of being very reactive, aldehydes are used for crosslinking natural polymers such as alginate, methylcellulose, chitosan-gelatin, collagen [15], gellan gum and xanthan gum [58].

### 3.3. Morphology Analysis

**Figure 4:**
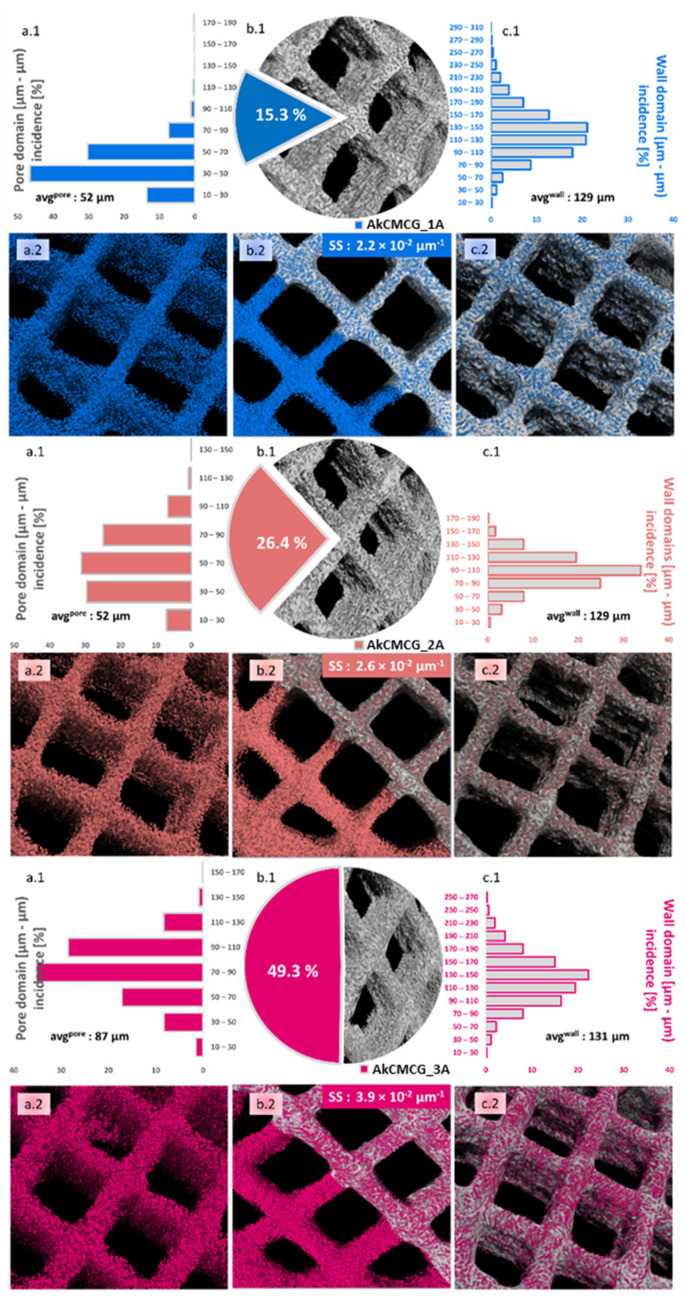
Quantitative and qualitative morphological analysis performed by µCT for the AkCMCG–1A, AkCMCG–2A and AkCMCG–3A 3D printed structures. Subsets (**a**) depict pore size distributions (**a.1**) and inverted tomograms of the pores (**a.2**); subsets (**b**) illustrate in (**b.1**) the ratio of pores vs. the sample (excluding the CAD model square shaped pores), and a dual representation of the overlapped tomograms of solids and empty spaces inside the lattice better used to comprehend the specific surface values (SS, calculated as the surface of the pores and of the outer area of the objects in µm^2^/total volume of the solid object in µm^3^) in (**b.2**), while (**c**) covers mostly the solid features in terms of wall thickness patterns (**c.1**) and the outer interface of open pores and exterior walls in the constructs (**c.2**).

µCT was employed to address how the AA variations impacted the morphology of the objects fabricated by the AkCMCG ink after freeze-drying. Both images and numerical values were extracted from the three tomograms, and the values are detailed in Figure 4. Annotations such as the mean values of specific surfaces (SS) are made on particular subdivisions of the figure.

The most obvious difference between the samples emerges from the calculated values of total porosity illustrated in the (c) subsections, where alongside the AA content used in the ink synthesis, porosity increases from 15.3% to 49.3%; this occurs probably because polymer chains interact with a solving environment where they can better loosen, therefore decreasing the interface tension during the phase separation occurring in the freezing stage of the pore patterning technique. As a result, solid matter can better dissociate from the solution. These values vary proportionally with the average pore size calculated as the weighted average from the histogram data (Figure 4a.1): 52→65→87 µm. The pore domain distribution falls under the incidence of Gaussian bells but changes from left skewed for AkCMCG-1A to right skewed for AkCMCG-3A; in the case of AkCMCG-2A, we observed the most balanced and flat distribution that also featured the least extended domains of empty spaces. Coupled with the fact that AkCMCG-2A also has the least wall size variation, with an average thickness of 108 µm, perhaps this particular AA concentration contributed to reaching an adequate solute:solvent ratio for a homogeneous morphological patterning.

As mentioned before, this is partly correlated with the wall thickness measurements in the AkCMCG-1A and AkCMCG-2A (Figure 4c.1) compositions, where the extension to which walls reach decreases. However, in the AkCMCG-3A object, where the mean values of both pores and walls are the highest, interesting phenomena occur: the total porosity almost doubles from the previous formulation, but so does the specific surface (that varies dependent on the AA content from 2.2 × 10^−2^→2.6 × 10^−2^ → 3.9 × 10^−2^ µm^−1^). It is believed that apart from the porosity itself, it is highly interconnective, and some topological features like surface roughness contributed to this increase.

In order to address this, Euler’s number was measured and used to assess connectivity density (Conn Dn in µm^−3^) and object surface density (S Dn in µm^−1^). Euler’s number is a basic topological measure that counts the number of independent entities, in these cases, voids and pores, completely surrounded by solid matter and the number of connections that need to be broken to divide the structure in two [72]. The percentage of closed porosity was extremely low in the three printed objects (<0.05%) and insufficient to explain the differences. Because the connectivity changes with the size of the object, this index is divided by the total volume and appropriately reported as the connectivity density. The object surface density also describes the density of superficial morphological features by reporting it to the studied volume. To better visualize these, Figure S3 from the Supplementary Material closely depicts the surface of the objects and the spatial distribution of the reconstructed pores under the detection limit of the equipment. Quantitatively, the S Dn varies between AkCMCG-1A and AkCMCG-2A from 2.2 to 2.36 µm^−1^ and reaches the value of 1.27 × 10^2^ µm^−1^ in the case of AkCMCG-3A, describing multiple features per unit; additionally, the Conn Dn values increased in the following order: 1.39 × 10^−4^ µm^−3^ for AkCMCG-3A, 2.47 × 10^−4^ µm^−3^ for AkCMG-1A and 2.51 × 10^−4^ µm^−3^ for AkCMCG-2A. Despite the small differences in Conn Dn, the important variations in the specific surface can be now justified.

**Figure 5:**
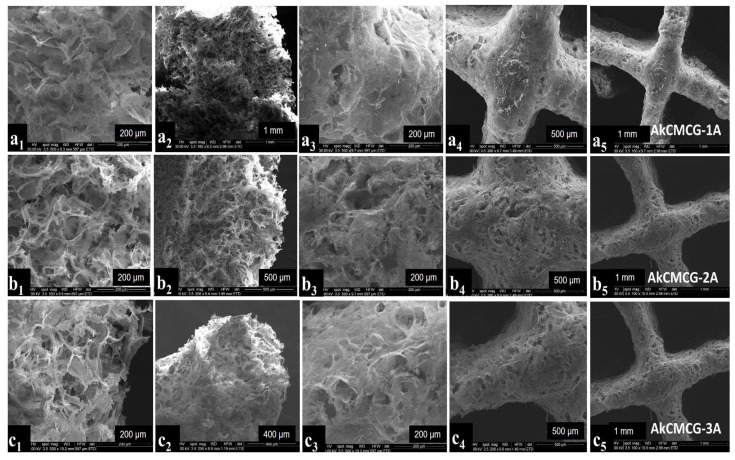
SEM images of the 3D printed layer-by-layer deposition of the AkCMCG formulation with different magnifications. The images of the AkCMCG-1A samples are denoted by (**a1**–**a5**); the images of the AkCMCG-2A samples are denoted by (**b1**–**b5**) and the images of the AkCMCG-3A samples are denoted by (**c1**–**c5**). Images denoted by (**a1**,**a2**,**b1**,**b2**,**c1**,**c2**) represent cross sections of the samples and the pictures denoted by (**a3**–**a5**,**b3**–**b5**,**c3**–**c5**) illustrate the top view of the surfaces of the specimens at 200 µm, 400 µm, 500 µm and 1mm.

SEM images allow the investigation of the morphology of the freeze-dried 3D printed scaffolds. The porous structure was confirmed by SEM as depicted in Figure 5.

All of the crosslinked samples exhibited a uniform distribution of the pores and a three-dimensional pore structure which verify a typical feature of natural polymer hydrogels. This particular structure is beneficial to cell development and nutrient migration in the body [11,68]. From looking at the SEM images, it was observed that the cross sections of the AkCMCG-1A 3D printed constructs presented fine walls while AkCMCG-2A and AkCMCG-3A showed a complex structure.

**Figure 6:**
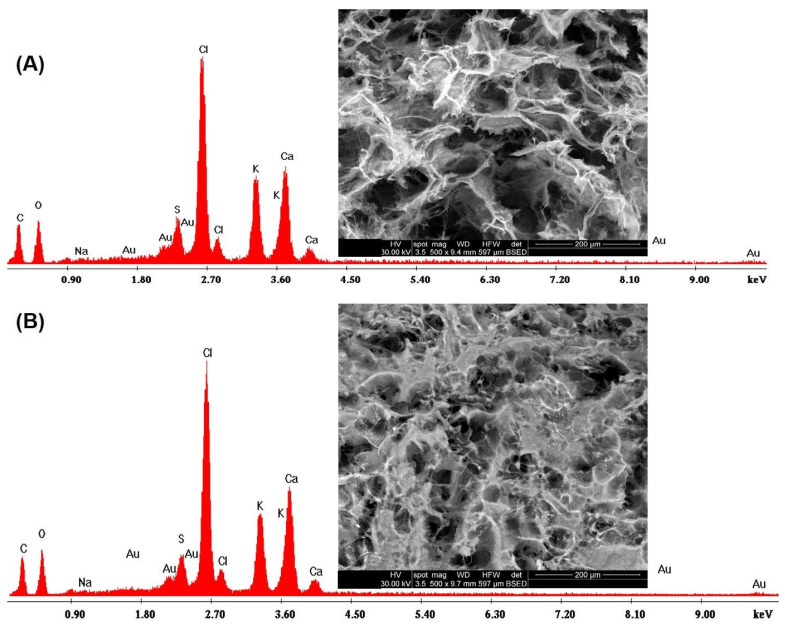
EDX analysis on (**A**) the cross section and on (**B**) the surface of the crosslinked samples.

Energy Dispersive X–Ray (EDX) analysis confirmed the presence of CaCl_2_ and KCl in the cross sections (Figure 6A) and on the surfaces (Figure 6B) of the scaffold matrix with the appearance of specific peaks of carbon, oxygen, sodium and sulfur.

### 3.4. Mechanical Properties of the 3D Construct

A well-known shortcoming of 3D hydrogel-based printed materials is their low mechanical strength, which limits their utility in a variety of therapeutic applications. In this research, the mechanical properties of the 3D printed Alg-kCG-CMC-GA scaffolds were evaluated through compression tests and subsequent computation of the Young’s modulus. The obtained values were correlated with the AA concentration of the GA solution in the crosslinking process. The results indicated that the stress resilience of the materials increases with an increase in AA concentration in the crosslinking mixture. This behavior is attributed to the higher number of activated –OH groups in the biopolymers’ structure that can interact with GA, in addition to the IPN induced by ionic interaction.

**Figure 7:**
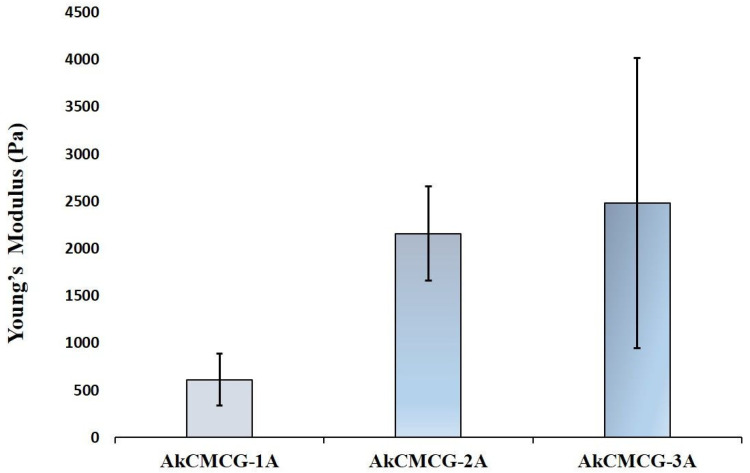
The compression modulus of the 3D printed hydrogel scaffold performed on rehydrated samples.

The 3D printed AkCMCG-3A deposition has the highest Young’s modulus (2.48 kPa ± 1.53), as the mechanical measurements reveal in Figure 7.

### 3.5. Swelling Behavior of the 3D Printed Hydrogel Structure

**Figure 8:**
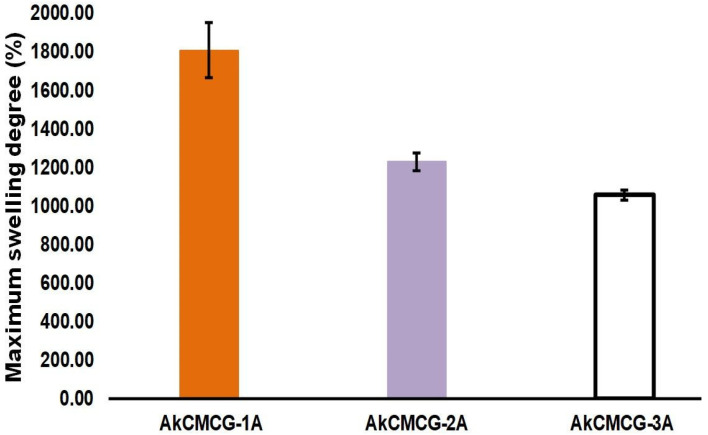
The maximum degree of swelling of the 3D printed scaffolds in PBS, pH = 7.4.

The biomaterials utilized in the medical field must have certain properties, such as water up-take capacity, that allow nutrient diffusion and maintain an optimal contact with the biological environment [73]. The rehydration ability of the dried 3D printed construct was assessed by the evaluation of the swelling rate (Figure 8).

From looking at Figure 8, it can be observed that the MSD is dictated by the increase in the AA concentration of the crosslinking solution. The samples immersed in the crosslinking mixture containing 1% AA had the highest maximum swelling degree (MSD = 1808.5% ± 143.2) when compared with the other two tested AA concentrations. Hence, by increasing the AA content in the crosslinking medium, GA can react with the available –OH groups of the three components from the printed scaffolds, increasing the hydrogen bonds of the hydrogels [4]. Therefore, this process generated a denser network, increasing the swelling equilibrium value and reducing the swelling rate. However, the AkCMCG-3A objects exhibited the highest average pore size and showed the lowest MSD, due to the thickness of the wall, as was demonstrated by the morphology analysis.

These findings were in agreement with the data collected from the mechanical testing; as the MSD decreases, the Young’s modulus increases. The highest Young’s modulus was 2.480 kPa ± 1.537 for the AkCMC-G3A scaffolds which reached the lowest maximum swelling degree (1057.17% ± 26.67) in 45 min, while the AkCMCG-1A samples, with the lowest Young’s modulus (0.613 kPa ± 0.272), recorded the highest MSD (MSD = 1808.5% ± 143.2) in a shorter time (15 min).

### 3.6. Structural Stability of the 3D Printed Scaffolds

Biodegradability is an important feature for scaffolds used for new tissue regeneration because it must be correlated with tissue growth for the construct to present enough stability when confronted with the mechanical stresses in the tissue environment [74].

**Figure 9:**
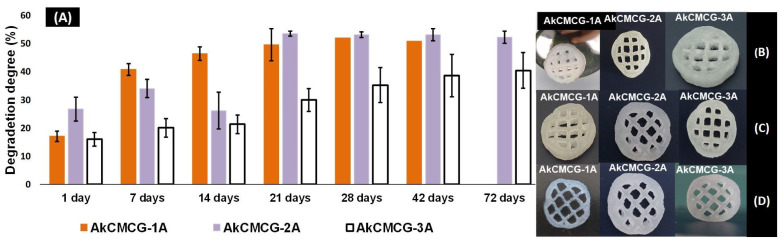
(**A**) In vitro degradation of AkCMCG hydrogels crosslinked with variable AA concentrations; (**B**) shows scaffold shape at 7 days in PBS, pH = 7.4; and (**C**) presents scaffold structure at 14 days and (**D**) at 28 days under the same conditions.

The stability of the dried 3D printed AkCMCG-1A, AkCMCG-2A and AkCMCG-3A scaffolds was studied also in PBS, pH = 7.4 at 37 °C over several days in order to evaluate the crosslinking efficiency. In this experiment, the degradation of the scaffolds over time, depicted in Figure 9, illustrated that the crosslinked samples degraded between 20% ± 3 and 41% ± 2 depending on the AA concentration at 7 days, while at 14 days, the 3D printed specimens exhibited a DD between 21% ± 3 and 46% ± 2 and at 72 days a DD of 40% ± 6 for AkCMCG-3A and 52% ± 2% for AkCMCG-2A.

The AkCMCG-3A printed constructs presented the highest stability; their trail period was up to 72 days, with a DD of 24.5% ± 6% at 7 days and 53% ± 6% at the end of the degradation test. These results can be associated with the increase in the acidic agent concentration in the crosslinking protocol, which may increase the hydrogen bond interaction between the polysaccharides and GA. This process could maintain the proximity of the polymer chains and reduce the degradation of the samples. The AkCMCG-3A samples were crosslinking in the mixture with the highest AA concentration, which led to a better crosslinking ability by obtaining a lower DD throughout the trial period. Moreover, these findings are affected by the porosity and the thickness of the walls of the sample. The mean values of both the pores and the thickness of the walls are the highest in the AkCMCG-3A scaffolds, as demonstrated by the µCT (Figure 4). Also, the scaffolds maintained their shape by the end of the experimental period as presented in Figure 9. In Figure 9B, the samples’ structures after 7 days immersed in PBS, pH = 7.4 are presented, and the samples’ structures are also shown in Figure 9C after 14 days subjected to PBS, pH = 7.4, and in Figure 9D at 28 days.

### 3.7. Drug Entrapment

The encapsulation efficiency (EE) of the VitB1 in the AkCMCG 3D printed samples crosslinked with different AA concentrations in the crosslinking mixture was assessed via UV spectroscopy at λ = 242 nm. The measurements were performed in triplicate.

The highest EE of VitB1 in AkCMCG was registered for the AkCMCG-3A sample at 12.14 ± 0.86%. For the AkCMCG-2A scaffold, the EE was 10.63 ± 0.29%, while the lowest EE was in AkCMCG-1A at 10.54 ± 2.56%. These results are a consequence of the samples’ degree of porosity, as was shown by morphology testing. As the degree of porosity increased (Figure 4), the EE of VitB1 was also higher. The VitB1 loading was performed by adsorbing the VitB1 solution from the outside of the wall to the inside of the lyophilized 3D printed structure through the pores of the sample. Moreover, the –NH_2_ and –OH groups from the vitamin structure (the zwitterion nature of VitB1) may interact with the hydrophilic functional groups available in the polysaccharide chains from which the scaffolds are composed, inducing hydrogen bond interactions that increase as the acidic concentration increases, especially for Alg, whose –COO^−^ groups are protonated to alginic acid under these conditions [75].

### 3.8. Fourier Transform Infrared (FT-IR) Spectroscopic Studies

The drug-loaded and neat 3D printed scaffolds were analyzed using FT-IR spectroscopy to obtain information about the structure of the IPN hydrogels.

**Figure 10:**
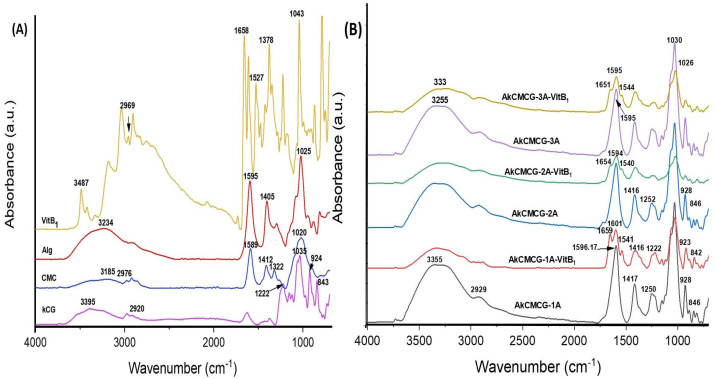
FT-IR spectra of (**A**) biopolymer powder and VitB1 powder, and (**B**) VitB1−loaded and unloaded three−component 3D printed samples.

Figure 10 displays the existence of functional groups of Alg, kCG, CMC and VitB1 along with the shifts and changes in these groups due to the interaction that occurs in the composition of the IPN materials.

The specific signals of Alg (Figure 10A) were confirmed at 2925 cm^−1^ for the aliphatic -CH symmetric stretching vibration, followed by = peaks at 1595 cm^−1^ and 1405 cm^−1^ for the asymmetric and symmetric stretching vibration of the –COO^−^ groups and at ~1025 cm^−1^ corresponding to the –C–O–C– ether bond [4,76]

The kCG characteristic bands were observed as follows: the signal at 2920 cm^−1^ corresponds to the saturated aliphatic –CH symmetric stretching vibration [26], the band at 1222 cm^−1^ is assigned to the ester sulfate (O=S=O) stretching vibration antisymmetric bond [77,78] and the bands at 842 cm^−1^ to the (–O–SO_3_) D-galactose-4-sulfate bond; at 924 cm^−1^ was the peak attributed to the –C–O–C– bond of 3,6-anhydrogalactase and at 1035 cm^−1^ the peak is generated by the –CH stretching from glycoside linkages of the polysaccharide structure [64,78].

The FT-IR spectra of the CMC (Figure 10A) depicted the following bands: the band at 2922 cm^−1^ was assigned to the –C–H stretching vibration of the methylene group, while the one at 1589 cm^−1^ is the characteristic band of the stretching absorption from the carbonyl group (–C=O); the peak with the shift at 1412 cm^−1^ was due to the symmetric stretching vibration of the –COO– carboxyl group of the salt form [68,79]. The band with moderate intensity at 1322 cm^−1^ was attributed to the symmetric deformation of –CH_2_ [76,79], and the strong one at 1020 cm^−1^ corresponds to the stretching vibration of the ether group –C–O–C–, the carbohydrate-specific linkage [26,80,81].

In the case of VitB1, the FT-IR spectra (Figure 10A) showed the characteristic peaks of the –OH and –NH_2_ groups at 3487 cm^−1^ [82], while the band at 2969 cm^−1^ was assigned to the methyl –C–H stretching [83] and the band at 1527 cm^−1^ showed aromatic –C=C– stretching [83,84]. The peaks at 1658 cm^−1^ and 1378 cm^−1^ were attributed to the –C=N bond and –C–N stretching and the band at 1043 cm^−1^ is due to the vibration of –C–O [82].

The FT-IR spectra of the 3D scaffolds (Figure 10B) confirmed the presence of polysaccharide-specific peaks and the carbonyl functional groups, as well as the band corresponding to the ester sulphate groups from the kCG, the glycosidic linkage, and the bands between 1300 cm^−1^ and 800 cm^−1^ known as the carbohydrate fingerprint region [78]. The peaks are slightly shifted, and additionally, a modification of the peak intensity was observed caused by the interactions that occurred between the biopolymers with the formation of IPN, the increase in hydrogen bonds and the occurrence of the cross-linking reaction [4,76,78,85].

As depicted in Figure 10B, for the VitB1-loaded samples, the FT-IR analysis proved the incorporation of the vitamin via the appearance of two additional peaks at 1651–1659 cm^−1^ corresponding to the C=N bond and at 1540–1544 cm^−1^ for the aromatic –C=C– stretching. A shifting of the band attributed to the stretching vibration of the –COO^−^ groups, which in the spectra’s plain 3D constructs do not occur, was also observed.

All of the FT-IR spectra displayed a broadband at 3000–4000 cm^−1^, corresponding to the stretching vibration of the -OH groups [4,81,83] and hydrogen bonding [26].

### 3.9. Dissolution Testing

**Figure 11:**
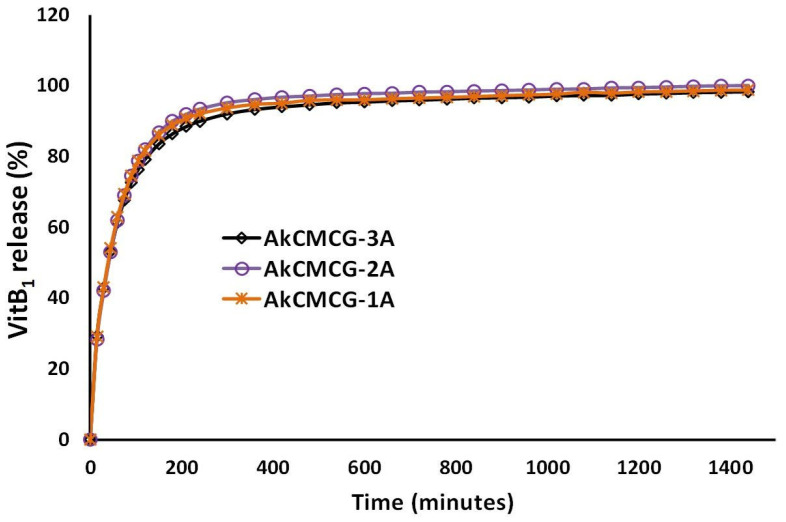
In vitro VitB1 release kinetics from AkCMCG-A3D scaffolds in PBS, pH = 7.4.

The release profile of the VitB1 from the AkCMCG-A 3D printed specimens was performed by placing the drug loaded scaffolds in the dissolution media, PBS, pH = 7.4; 37 °C and is presented in Figure 11.

As can be observed from looking at Figure 11, all three 3D printed scaffolds exhibited a burst release of the vitamin for 30 min. Even though the EE is different for each 3D printed object, the drug is being released from the IPN scaffolds in the same manner. In the first 15 min, in PBS, a burst release of the VitB1 (29% for both AkCMCG-1A and AkCMCG-3A and 28% for AkCMCG-2A) is detected due to the release of the drug molecules entrapped near the samples’ surface [16]. After 2 h, the released amount was found to be 79% in the AkCMCG-1A sample and around 82% in AkCMCG-2A and AkCMCG-3A. After 4 h (210 min), the resealed curve reached a plateau and the amount of VitB1 released was 90% in AkCMCG-1A, 92% in AkCMCG-2A and 93% in AkCMCG-3A. The release of the therapeutic substance from the AkCMCG-A–printed formulations can be promoted by the internal electrostatic repulsive interactions that occurred between the partially negatively charged dense COOH groups of the Alg and CMC chains and the negative sulfate groups of the kCG in the alkaline environment, which favored the release of the vitamin [16].

## 4. Conclusions

In this research, a new ink formulation prepared using three natural polymers, Alg, kCG and CMC, and GA was successfully developed in order to test its printability for an extrusion-based 3D printing platform. The scaffolds were ionically crosslinked using CaCl_2_, KCl and GA in an acidic solution, obtaining the IPN which was confirmed by FT-IR spectra. The influence of the acetic acid concentration in the crosslinking mixture on the structural stability of the printed samples was assessed. The increasing of the AA concentration enhanced the swelling equilibrium value, reduced the swelling rate and increased the stability of the samples in PBS, pH = 7.4. The samples crosslinked in the mixture containing 1% AA had the highest maximum swelling degree (MSD = 1808.5 ± 143.2%). In vitro degradation of the AkCMCG hydrogels cross-linked with varying AA concentrations showed that the AkCMCG-3A samples presented the highest stability, up to 72 days, with a DD of 24.5 ± 6% at 7 days and 53 ± 6% at the end of the degradation test. The mechanical test showed that the 3D printed AkCMCG-3A deposition had the highest Young’s modulus. According to the morpho-structural characterization, the obtained freeze-dried 3D printed samples exhibited printing fidelity and homogenous and porous structures suitable to cell development. The AkCMCG-3A samples displayed the thickest walls and an enhanced porosity, up to 49.3%, as the µCT analysis showed. VitB1 encapsulation efficiency in the scaffolds was estimated by UV-Vis spectroscopy and confirmed by FT-IR; the highest encapsulation was 12.14 ± 0.86% in the AkCMCG-3A 3D printed objects. The release kinetics of VitB1 showed the same release behavior in all three types of specimen with a burst release of approximately 29% in the first 15 min. The approach of this research suggested that the proposed Alg-kCG-CMC-GA scaffolds could be a useful option for pharmaceutical encapsulation and in tissue engineering.

## Figures and Tables

**Table 1:** Printing parameters for optimization.

Material	Printing Parameters			Scaffold Characteristics		
	Extrusion Pressure (kPa)	Printing Speed (mm/s)	Inner Diameter	Scaffold Diameter (mm)	Number of Layers	Line Space (mm)
AkCMCG	195–200	6	0.20	14	2	3.5
	195–200	6	0.20	14	14	3.5
	180–185	4	0.25	14	2	3.5
	180–185	6	0.25	14	2	3.5
	160–165	4	0.25	14	14	3.5
	165	4	0.25	14	16	3.5
	160–165	4	0.25	14	40	3.5
	160–165	4	0.25	14	60	3.5
	175	7	0.25	9	4	1.5
	175	7	0.25	9	4	1.75
	180	4	0.25	9	16	1.75
	170	7	0.25	9	16	1.80

## Data Availability

Data are contained within the article.

## Supplementary Materials

The following supporting information can be downloaded at: https://www.mdpi.com/article/10.3390/polym16111592/s1, Figure S1: Macroscopic imagines of various printed scaffolds. The objects with 14mm in diameter and line space of 3.5 mm of the grid cylinder, presented in A has 2 layers and the one in B has 14 layers, were printed using a needle with the inner diameter of 0.20 mm and the same the printing speed 6 mm/s and the same extrusion pressure 195–200 kPa. The structure C with 2 layers, was printed using a 180-185 kPa while, the constructs D (14 layers) and E (16 layers) were extruded at 160–165 kPa from a 0.25 inner diameter needle with a printing speed of 4 mm/s. A 60-layer grid cylinder with 3.5 mm line space printed using an 4 mm/s printing speed and a 160–165 kPa pressure is displayed in Figure S2F. The scaffolds with 9 mm in diameter and 4 layers presented in Figure S2G (1.5 mm line space) and S2H (1.75 mm line space) were manufactured using an 7 mm/s printing speed and 175 kPa pressure. A 16-layer designs with 9 mm in diameters, were extruded at 180 kPa with 4 mm/s for I and at 170 kPa with 7 mm/s for J. The line space of the grid was 1.75 mm for I and 1.80 mm for H; Figure S2: Macroscopic imagines of continuous and homogenous ink filament (A) ink filament; (B) side view of a 14 layers scaffold; (C), of a 16 layers scaffold; (D) and of a 60 layers 3D printed scaffold; Figure S3: Close-ups of the AKCMC-1AA, AKCMC-2AA and AKCMC-3AA depicting in the *i.* subdivisions the interconnectivity and density of reconstructed porosity (inverted tomogram) and in the *ii.* sets the coarser/finer topographical particularities of the 3D printed objects, in strong correlations to the Conn Dn and S Dn measurements discussed in the main body of the manuscript.
