# Supplementary material for: Fabrication of k-Carrageenan/Alginate/Carboxymethyl Cellulose basedScaffolds via 3D Printing for Potential Biomedical Applications
Source: Polymers (Basel). 2024 Jun 4;16(11):1592. doi: 10.3390/polym16111592 (PMC11174997; doi:10.3390/polym16111592)
Supplement: Supplementary file 1 [file polymers-16-01592-s001.zip › polymers-3006600-supplementary.pdf]

polymers

MDPI

Supplementary Material

Fabrication of k-carrageenan/alginate/carboxymethyl cellulose scaffolds made by 3D printing for potential biomedical application

Cristina Stavarache [1,2,] Adi Ghebaur [1], Andrada Serafim [1], George Vlăscanu [1,3], Eugeniu Vasile [4], Sorina Alexandra Gârea [1] and Horia Iovu [1,5]*

[1] Advanced Polymer Materials Group, National Polytechnic University of Science and Technology Bucharest, 1-7 Gh. Polizu Street, 011061, Bucharest, Romania,
[2] C.D. Nenițescu" Institute of Organic and Supramolecular Chemistry, 202-B Spl. Independentei, RO-060023, Bucharest, Romania
[3] Faculty of Medical Engineering, National University for Science and Technology POLITEHNICA Bucuresti, 1-7 Gh. Polizu Street, 011061 Bucharest, Romania
[4] Department of Science and Engineering of Oxide Materials and Nanomaterials, Faculty of Applied Chemistry and Material Science, National Polytechnic University of Science and Technology Bucharest, 1-7 Gh. Polizu Street, 011061, Bucharest, Romania
[5] Academy of Romanian Scientists, 54 Splaiul Independentei, 050094, Bucharest, Romania
* horia.iovu@upb.ro

Scaffolds fabrication

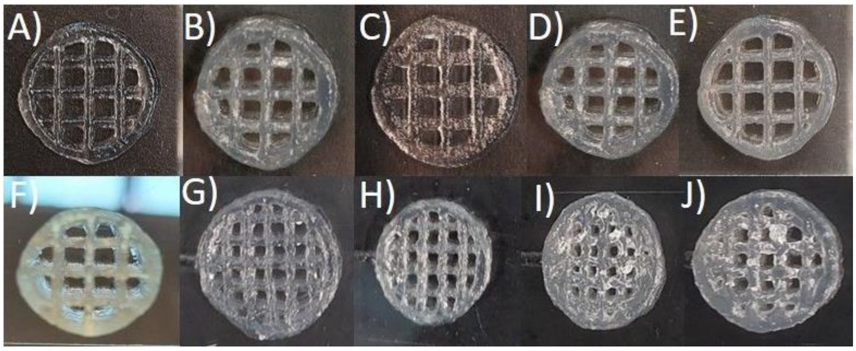

Figure S1. Macroscopic imagines of various printed scaffolds. The objects with 14mm in diameter and line space of 3.5 mm of the grid cylinder, presented in A has 2 layers and the one in B has 14 layers, were printed using a needle with the inner diameter of 0.20 mm and the same the printing speed 6 mm/s and the same extrusion pressure 195-200 kPa. The structure C with 2 layers, was printed using a 180-185 kPa while, the constructs D (14 layers) and E (16 layers) were extruded at 160-165 kPa from a 0.25 inner diameter needle with a printing speed of 4 mm/s. A 60-layer grid cylinder with 3.5 mm line space printed using an 4 mm/s printing speed and a 160-165 kPa pressure is displayed in Figure S2 F. The scaffolds with 9 mm in diameter and 4 layers presented in figure S2 G (1,5 mm line space) and S2 H (1.75 mm line space) were manufactured using an 7 mm/s printing speed and 175 kPa pressure. A 16-layer designs with 9 mm in diameters, were extruded at 180 kPa with 4 mm/s for I and at 170 kPa with 7 mm/s for J. The line space of the grid was 1.75 mm for I and 1.80 mm for H.

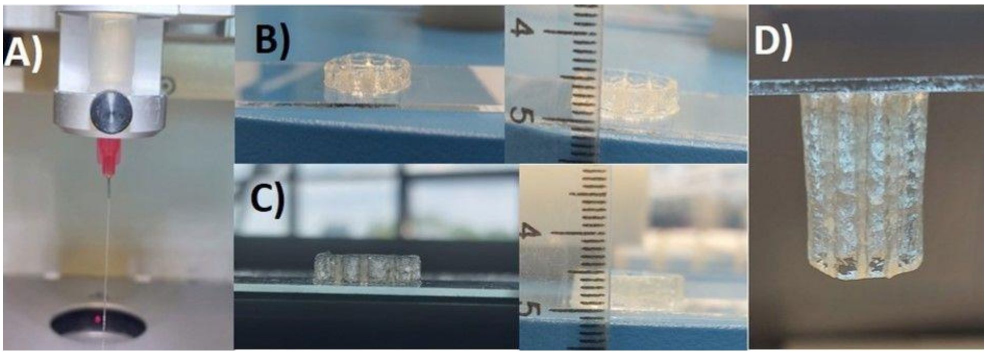

Figure S2. Macroscopic imagines of continuous and homogenous ink filament A) ink filament; B) side view of a 14 layers scaffold; C), of a 16 layers scaffold; D) and of a 60 layers 3D printed scaffold.

Morphological Analysis

Morpho-structural characterization of 3D printed scaffolds was carried out through micro-CT (μCT)

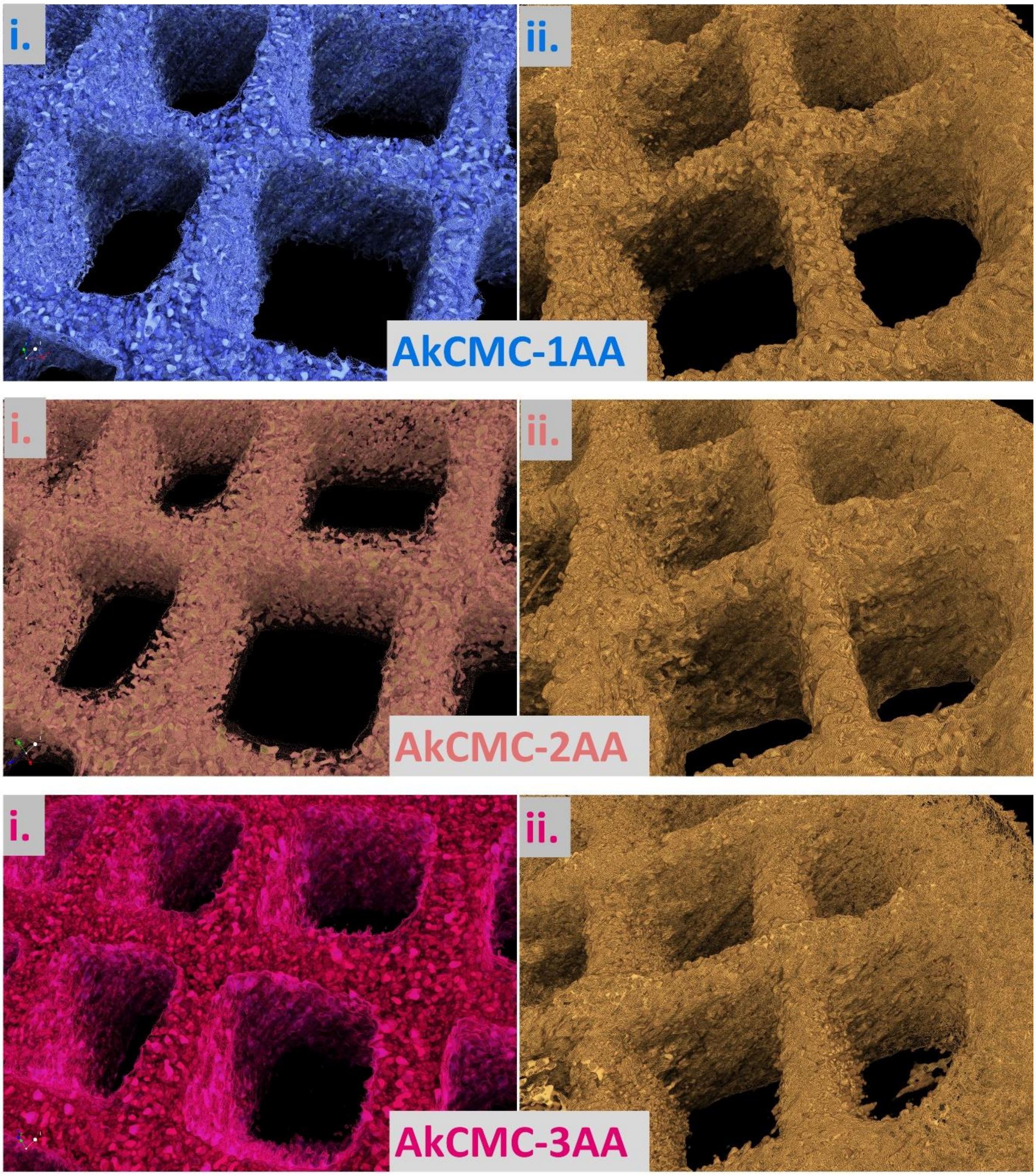

Figure S3. Close-ups of the AKCMC-1AA, AKCMC-2AA and AKCMC-3AA depicting in the *i.* subdivisions the interconnectivity and density of reconstructed porosity (inverted tomogram) and in the *ii.* sets the coarser/finer topographical particularities of the 3D printed objects, in strong correlations to the Conn Dn and S Dn measurements discussed in the main body of the manuscript.
